# A Comprehensive Review on Comparative Analysis of Operative Efficiency and Postoperative Recovery in Robotic Versus Laparoscopic Hepatectomy

**DOI:** 10.7759/cureus.67262

**Published:** 2024-08-20

**Authors:** Mohammed Azeem Khan, Chandrashekhar Mahakalkar, Shivani Kshirsagar, Simran Dhole, Sparsh Dixit

**Affiliations:** 1 General Surgery, Jawaharlal Nehru Medical College, Datta Meghe Institute of Higher Education and Research, Wardha, IND

**Keywords:** comparative analysis, postoperative recovery, operative efficiency, minimally invasive liver surgery, laparoscopic hepatectomy, robotic hepatectomy

## Abstract

Minimally invasive liver surgery, particularly hepatectomy, has evolved significantly with the advent of laparoscopic and robotic techniques. These approaches offer potential benefits over traditional open surgery, including reduced postoperative pain, shorter hospital stays, faster recovery, and improved cosmetic outcomes. This comprehensive review aims to compare the operative efficiency and postoperative recovery outcomes of robotic and laparoscopic hepatectomy. It seeks to provide an in-depth analysis of the advantages and limitations of each technique, assess their cost-effectiveness, and explore emerging trends and future directions in minimally invasive liver surgery. A comprehensive literature search was conducted to identify studies comparing robotic and laparoscopic hepatectomy. The review includes an analysis of operative time, intraoperative blood loss, conversion rates, postoperative pain, length of hospital stay, complication rates, oncological outcomes, and overall cost. Additionally, advancements in technology and future research directions were explored to provide a comprehensive overview of the current landscape and future potential of these surgical techniques. Both robotic and laparoscopic hepatectomy have demonstrated comparable outcomes in terms of oncological safety and effectiveness. However, robotic hepatectomy offers advantages in terms of precision and dexterity, particularly in complex cases, due to its advanced visualization and instrumentation. Laparoscopic hepatectomy, while associated with shorter operative times and lower costs, is limited by technical challenges, especially in major liver resections. The review also highlights the increasing adoption of robotic systems, despite their higher costs and the need for specialized training. Robotic and laparoscopic hepatectomy are both viable options for minimally invasive liver surgery, each with distinct advantages and limitations. The choice between the two should be based on patient-specific factors, the complexity of the procedure, and the surgeon's expertise. Ongoing advancements in technology, including the integration of artificial intelligence and augmented reality, are expected to further refine these techniques, enhancing their efficacy and accessibility. Future research should focus on large-scale, multicenter trials to provide more definitive comparisons and guide clinical decision-making.

## Introduction and background

Liver surgery, particularly hepatectomy, plays a crucial role in the management of various liver diseases, including primary and metastatic liver tumors, benign liver lesions, and certain non-neoplastic conditions [1]. Traditionally, open surgery has been the standard approach for liver resections; however, advancements in surgical technology and techniques have led to the emergence of minimally invasive options. These include laparoscopic and robotic hepatectomy, both of which offer potential benefits over open surgery, such as reduced postoperative pain, shorter hospital stays, faster recovery times, and improved cosmetic outcomes [2]. Minimally invasive techniques in liver surgery have gained significant attention due to their ability to minimize the physiological impact of surgery while maintaining oncological safety and efficacy. The adoption of these techniques has been driven by the desire to enhance patient outcomes and improve the overall surgical experience. As a result, laparoscopic and robotic hepatectomy have become increasingly popular, offering patients less invasive alternatives to traditional open liver surgery [3].

Laparoscopic hepatectomy, the first minimally invasive approach to be widely adopted in liver surgery, involves the use of small incisions and specialized instruments to perform the procedure. It has been associated with reduced blood loss, fewer complications, and a quicker return to normal activities compared to open surgery. However, the complexity of liver anatomy and the technical challenges associated with laparoscopic surgery can limit its application, particularly for major liver resections [4]. Robotic hepatectomy, an advanced minimally invasive technique, has emerged as a promising alternative to laparoscopic surgery. The robotic platform offers several advantages, including enhanced dexterity, precision, and visualization through a 3D high-definition camera. These features can potentially overcome some of the limitations of laparoscopic surgery, allowing for more complex resections with greater ease. Despite these benefits, the adoption of robotic hepatectomy has been slower due to factors such as high costs, limited availability of robotic systems, and the need for specialized training [5].

The primary objective of this comprehensive review is to compare the operative efficiency and postoperative recovery outcomes of robotic and laparoscopic hepatectomy. By analyzing current literature and clinical data, this review aims to provide a detailed examination of both techniques, highlighting their respective advantages, limitations, and areas for improvement. Additionally, the review seeks to explore the cost-effectiveness of these minimally invasive approaches and discuss emerging trends and future directions in the field of liver surgery. Through this analysis, the review intends to offer valuable insights for surgeons, healthcare providers, and patients, aiding in the decision-making process for selecting the most appropriate surgical approach for liver resections.

## Review

Overview of hepatectomy techniques

Laparoscopic Hepatectomy

Laparoscopic hepatectomy (LH) was first successfully performed in 1991, marking the beginning of its development in the early 1990s [6]. Over the years, the technique has evolved significantly, driven by advancements in surgical instruments and methodologies. Initially, laparoscopic procedures were limited to simple resections, but as surgeons gained experience and confidence, the technique expanded to include more complex procedures, such as major hepatectomies. This progression has made LH a viable option for a broader range of patients with liver lesions [7]. The indications for laparoscopic hepatectomy include benign liver lesions, such as hemangiomas and focal nodular hyperplasia, as well as malignant tumors, particularly small hepatocellular carcinomas, and metastatic liver diseases [8]. Additionally, LH is often considered for patients who have undergone previous abdominal surgeries, as a laparoscopic approach can minimize additional trauma. However, contraindications include large tumors (typically over 5 cm), severe liver dysfunction (such as cirrhosis or portal hypertension), and anatomical challenges that may complicate laparoscopic access or visibility [9]. The surgical technique for laparoscopic hepatectomy involves several key steps. First, the patient is positioned in a supine or lateral decubitus position. Trocar placement follows, providing access to the camera and surgical instruments. Laparoscopic dissection is then performed using specialized instruments designed to minimize blood loss [10]. Finally, the specimen is extracted, often through a larger port or a mini-laparotomy. The equipment used in LH includes laparoscopic graspers, scissors, energy devices (such as harmonic scalpels), and a high-definition camera system to enhance visualization during the procedure [11].

Robotic Hepatectomy

Robotic hepatectomy (RH) has gained significant traction since the introduction of robotic surgical systems in the early 2000s [12]. The adoption of this technique has been driven by the benefits of robotic technology, including enhanced dexterity, three-dimensional visualization, and ergonomic advantages for surgeons. As robotic systems have become more widespread, their use in liver surgery has expanded, enabling complex resections that were previously difficult to achieve with traditional laparoscopic techniques [13]. The indications for robotic hepatectomy closely mirror those for laparoscopic approaches, including malignant liver tumors, particularly in challenging anatomical locations, and benign lesions where minimally invasive techniques are preferred. However, specific contraindications for RH include large or locally advanced tumors that may necessitate extensive resection, severe comorbidities that could complicate anesthesia or recovery, and limited access due to prior surgeries or anatomical anomalies [14]. The surgical technique for robotic hepatectomy involves several key steps. The patient is positioned, similarly to laparoscopic procedures, often with the aid of a robotic cart. Following trocar placement, the robotic arms are docked to the patient. Surgical dissection is then performed using robotic instruments with enhanced motion and precision. The specimen is typically removed through an enlarged port or mini-laparotomy. The essential equipment for RH includes the robotic surgical system (e.g., da Vinci Surgical System), which features robotic arms, a console for the surgeon, and a high-definition 3D camera to provide detailed visualization throughout the procedure [15].

Comparative analysis of operative efficiency

A comparative analysis of operative efficiency between robotic and laparoscopic hepatectomy highlights several key factors affecting surgical outcomes. One of the most significant differences is operative time [16]. Robotic hepatectomy generally involves a longer average operative time compared to laparoscopic procedures. For example, median operative times for robotic surgeries are approximately 285 minutes, while laparoscopic surgeries average around 147 minutes [5]. This disparity can be attributed to factors such as the complexity of the procedure, setup time for the robotic system, and the surgeon's familiarity with the robotic platform. Additionally, variations in defining operative time across studies - particularly regarding setup and docking times - can complicate direct comparisons [17]. Another critical area of comparison is blood loss and transfusion requirements. Robotic hepatectomy is often associated with lower estimated blood loss compared to laparoscopic techniques [18]. Studies have shown a median blood loss of 100 mL for robotic procedures, whereas laparoscopic surgeries report approximately 300 mL. The reduced blood loss in robotic procedures is likely due to robotic systems' superior visualization and precision. Consequently, the need for blood transfusions tends to be lower in robotic cases, though exact rates can vary based on the complexity of the surgical cases and the surgeon’s experience [19]. Conversion rates to open surgery are also an important metric for assessing operative efficiency. Robotic hepatectomy generally shows lower conversion rates compared to laparoscopic techniques. Some studies report no conversions in robotic cases, while laparoscopic procedures have conversion rates as high as 23% [20]. This trend suggests that robotic techniques may provide a more stable approach in complex cases, potentially reducing the need for conversion to open surgery. Conversion often occurs due to complications or unexpected findings during the procedure. Factors such as liver anatomy complexity, tumor size, and the surgeon's expertise with the technique significantly influence these rates. Robotic systems' enhanced visualization and maneuverability may help address some of these challenges [21]. Lastly, the surgeon's learning curve and expertise are crucial to robotic and laparoscopic hepatectomy outcomes. Research indicates that as surgeons gain experience with robotic systems, operative times generally decrease, and complication rates may improve. Proficiency in robotic surgery requires specific training and familiarity with the robotic platform, and it is beneficial for surgeons to be also skilled in laparoscopic techniques, as many skills are transferable. Institutions often emphasize the importance of structured training programs to improve surgical outcomes in robotic hepatectomy [22].

Comparative analysis of postoperative recovery

RH and LH exhibit similar outcomes regarding postoperative pain levels and analgesic requirements. There is no significant difference in the length of hospital stay between RH and LH (mean difference = 0.10 days, 95% CI: -0.38 to 0.58, p = 0.69), and morbidity rates, which are likely related to postoperative pain, are comparable between the two techniques [23]. Multimodal analgesia regimens, which include opioids, non-opioids, and non-pharmacologic methods, are recommended for managing postoperative pain, with specific components tailored to individual patients and procedures [24]. The duration of hospitalization is similar for both RH and LH, with no significant difference in the length of stay. Factors influencing discharge timing typically include effective pain management, return of bowel function, ability to tolerate oral intake, and mobility independence [25]. Postoperative complications and their management were also comparable between RH and LH, with similar morbidity rates. Inadequate pain control can increase the risk of complications such as pneumonia by impairing deep breathing and coughing, emphasizing the importance of prompt recognition and treatment of complications for optimal recovery [26]. Both techniques show comparable timelines for functional recovery and quality of life. Patients experiencing poorly controlled pain may be less inclined to mobilize, potentially delaying functional recovery and rehabilitation. Multimodal analgesia, incorporating non-pharmacologic therapies like exercise, relaxation techniques, and cognitive-behavioral therapy, can support enhanced recovery [27]. Given the similar pain levels, hospital stay durations, and complication rates, patient-reported outcomes and quality of life measures are likely similar between RH and LH. In summary, robotic and laparoscopic hepatectomy offer comparable outcomes regarding postoperative pain, hospital stay length, complication rates, and functional recovery, providing similar postoperative experiences for patients undergoing liver resection [2].

Oncological outcomes

When comparing RH and LH, studies indicate that both techniques achieve comparable rates of R0 (complete) resection margins, reflecting the oncological safety of each approach. Systematic reviews have found no significant differences in R1 (incomplete) resection rates between RH and LH, suggesting that both methods effectively ensure complete tumor removal [28]. Additionally, long-term survival outcomes, including overall and disease-free survival, appear similar for patients undergoing RH or LH. Research indicates comparable recurrence rates for hepatocellular carcinoma between the two techniques, reinforcing that the surgical approach does not negatively impact oncological outcomes [29]. The adequacy of lymph node dissection during hepatectomy is crucial for accurate staging and prognosis in liver malignancies. While both RH and LH can achieve adequate lymph node retrieval, specific data directly comparing these techniques is somewhat limited. Most studies have concentrated on the completeness of tumor resection rather than lymph node outcomes [30]. However, it is well-established that thorough lymphadenectomy contributes positively to staging accuracy and may improve long-term outcomes. Although the specific advantages of robotic versus laparoscopic techniques regarding lymph node dissection are not yet fully clarified, it is generally accepted that effective lymph node retrieval is essential for optimal patient management [31].

Cost-effectiveness analysis

When assessing the cost-effectiveness of robotic versus laparoscopic hepatectomy, it is crucial to consider both direct and indirect costs and the broader economic implications for the healthcare system and patients. This analysis provides a comprehensive overview of these factors [32]. Direct costs associated with surgical procedures include various elements, such as surgical fees and consumables. Robotic hepatectomy generally incurs higher initial costs due to the expense of robotic systems, which can range from $1.5 million to $2.5 million, with annual maintenance costs potentially exceeding $100,000 [32]. In contrast, laparoscopic procedures typically involve lower direct costs, primarily due to the less expensive standard laparoscopic instruments and equipment. The overall cost for laparoscopic setups is estimated to be 30% to 50% lower than that of robotic systems. Furthermore, robotic procedures often require specialized single-use instruments, adding between $1,500 and $3,000 per procedure, while laparoscopic procedures usually use reusable instruments, reducing costs over time [33]. Indirect costs are also a significant factor in the cost comparison. The length of hospital stay following surgery is crucial in determining overall costs. Robotic hepatectomy may result in shorter hospital stays due to potentially lower complication rates, which can help offset some of the higher initial costs [21]. Studies indicate that patients undergoing robotic surgery may experience a reduction in hospital stay by one to two days compared to those undergoing laparoscopic procedures. Additionally, shorter recovery times can lead to decreased indirect costs related to lost productivity for both patients and caregivers [34]. Postoperative care needs, including follow-up visits and potential readmissions, should also be considered. If robotic surgery results in fewer complications, the long-term indirect costs may be lower, enhancing its cost-effectiveness [35]. The economic implications of robotic versus laparoscopic hepatectomy extend beyond individual procedures, impacting the healthcare system as a whole. The higher upfront costs of robotic systems can strain hospital budgets, particularly in resource-limited settings [36]. However, if robotic surgery leads to fewer complications and shorter recovery times, the overall burden on the healthcare system may be mitigated. Some studies suggest that the long-term savings associated with robotic surgery - due to reduced readmission rates and complications - can offset the higher initial costs, leading to more efficient healthcare resource allocation [36]. For patients, the financial implications of these surgical options can vary significantly based on insurance coverage. Robotic surgeries may entail higher out-of-pocket costs due to elevated deductibles and copayments associated with advanced surgical techniques. This financial burden can be particularly challenging for patients, potentially leading to disparities in access to robotic surgery based on socioeconomic status. While the initial costs of robotic surgery may be higher, improved postoperative recovery and reduced complications can enhance the quality of life outcomes, potentially justifying the increased expenses from a patient-centered perspective [37].

Emerging trends and future directions

**Figure 1:**
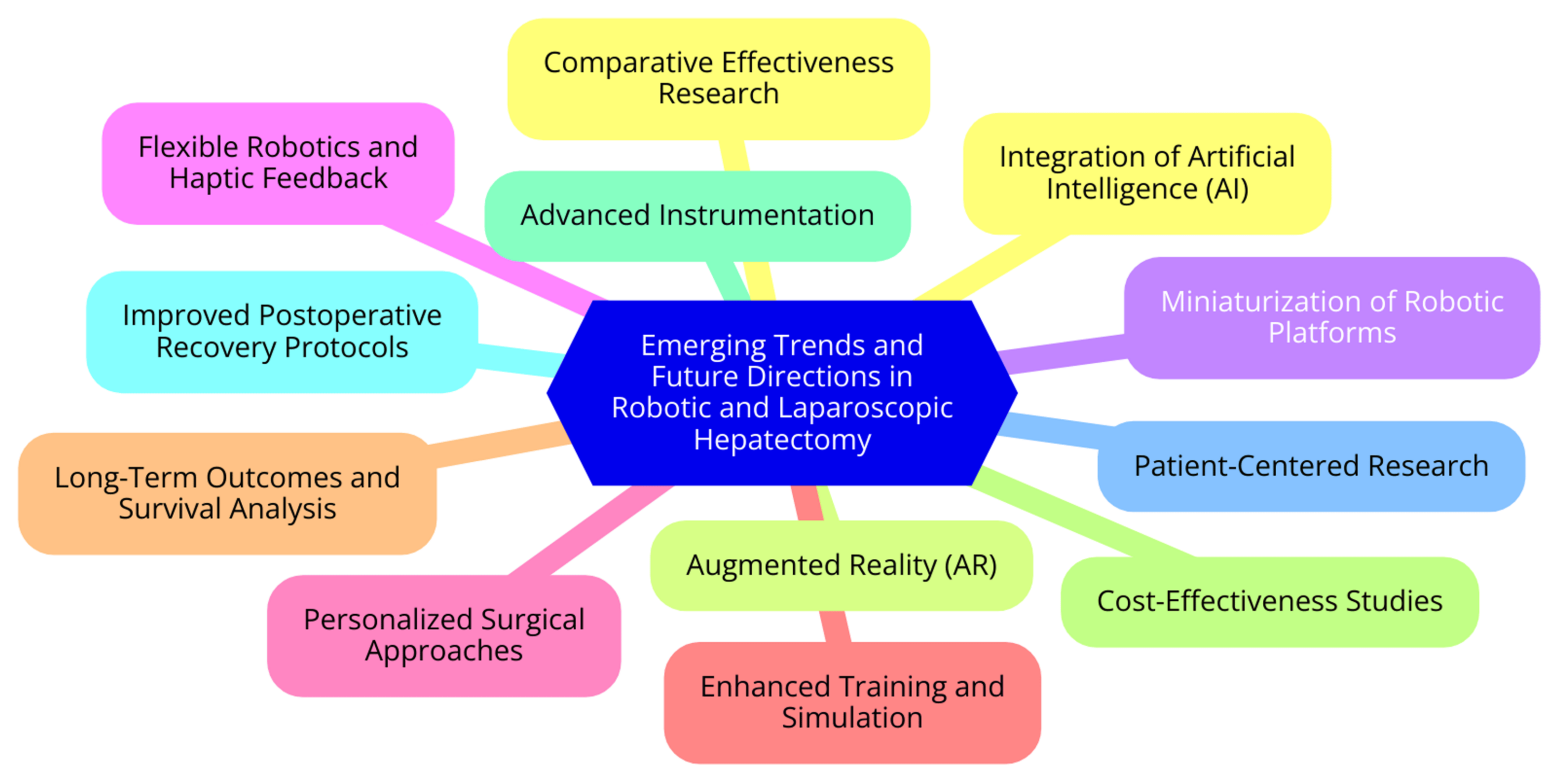
Emerging trends and future directions in robotic and laparoscopic hepatectomy Image Credit: Dr Mohammed Azeem Khan

Robotic and laparoscopic surgical systems are experiencing significant technological advancements aimed at enhancing precision, efficiency, and patient outcomes. Innovations such as the integration of artificial intelligence (AI) are transforming surgical practices by enabling real-time analysis of the extensive visual data generated during procedures. This capability can assist surgeons in making informed decisions during surgery, potentially improving outcomes [38]. Additionally, augmented reality (AR) is emerging as a powerful tool for enhancing visualization of intraoperative anatomy, allowing surgeons to navigate complex structures with greater accuracy. Moreover, the miniaturization of robotic platforms is reducing the surgical footprint, making these systems more versatile and easier to integrate into various surgical environments. Flexible robotics and haptic feedback technologies are also being developed to enhance dexterity and provide tactile sensations, further improving the surgeon's ability to perform intricate maneuvers. Collectively, these technological innovations have the potential to narrow the gap between robotic and laparoscopic approaches in terms of operative efficiency and postoperative recovery [39]. As robotic and laparoscopic techniques continue to evolve, there is an increasing emphasis on personalized surgery and careful patient selection for each approach. Establishing clear criteria for choosing robotic and laparoscopic methods is essential for optimizing surgical outcomes [40]. Factors to consider include tumor size and location, the extent of liver resection required, and the presence of patient comorbidities or risk factors. Additionally, the surgeon's experience and preferences play a crucial role in determining the most appropriate surgical technique. By customizing the surgical approach based on these individual patient factors, surgeons can enhance the likelihood of successful outcomes and minimize complications, ultimately improving patient satisfaction [40]. Despite the growing body of evidence supporting robotic and laparoscopic hepatectomy, several knowledge gaps warrant further investigation. Future research should focus on conducting large, randomized controlled trials to directly compare the two techniques, validating the existing findings and clarifying their relative advantages. Evaluating long-term oncologic outcomes and survival rates for patients undergoing liver resection for malignancy is critical to understanding the broader implications of each approach [5]. Additionally, assessing the cost-effectiveness of robotic versus laparoscopic techniques, including both surgical and postoperative costs, will be vital for informed healthcare decision-making. Finally, investigating the impact of surgeon learning curves on outcomes for each technique can provide valuable insights into how experience influences surgical success. Addressing these areas through well-designed clinical studies will help guide the future adoption and implementation of robotic and laparoscopic hepatectomy in routine surgical practice, ultimately benefiting patients and healthcare systems alike [41]. Emerging trends and future directions in robotic and laparoscopic hepatectomy are shown in Figure 1.

## Conclusions

In conclusion, the comparative analysis of robotic and laparoscopic hepatectomy reveals distinct advantages and limitations for each technique, highlighting the nuanced decision-making required in selecting the most appropriate surgical approach. While laparoscopic hepatectomy has established itself as a minimally invasive option with a solid track record of safety and efficacy, robotic hepatectomy offers enhanced precision and dexterity, particularly for complex liver resections. Both techniques demonstrate comparable outcomes in terms of operative efficiency and postoperative recovery, although the choice between them may depend on factors such as surgeon expertise, patient-specific considerations, and the availability of technology. The cost-effectiveness of robotic surgery remains a topic of ongoing debate, with high initial expenses balanced against potential long-term benefits. As technological advancements continue to evolve, including the integration of artificial intelligence and improved imaging modalities, the future of minimally invasive liver surgery promises further refinement and expansion of these techniques. Ultimately, a personalized approach, considering both the surgeon's skill set and the patient's needs, will remain essential in optimizing surgical outcomes and enhancing the overall patient experience.
